# Abnormal body composition is a predictor of adverse outcomes after autologous haematopoietic cell transplantation

**DOI:** 10.1002/jcsm.12570

**Published:** 2020-03-25

**Authors:** Saro H. Armenian, Aleksi Iukuridze, Jennifer Berano Teh, Kristen Mascarenhas, Alex Herrera, Jeannine S. McCune, Jasmine M. Zain, Sogol Mostoufi‐Moab, Shana McCormack, Thomas P. Slavin, Jessica M. Scott, Lee W. Jones, Can‐Lan Sun, Stephen J. Forman, F. Lennie Wong, Ryotaro Nakamura

**Affiliations:** ^1^ Department of Population Sciences City of Hope Duarte CA USA; ^2^ Department of Hematology and Hematopoietic Cell Transplantation City of Hope Duarte CA USA; ^3^ Department of Pediatrics Children's Hospital of Philadelphia Philadelphia PA USA; ^4^ Department of Medicine Memorial Sloan Kettering Cancer Center New York NY USA; ^5^ Department of Medicine Weill Cornell Medical Center New York NY USA; ^6^ Department of Supportive Care Medicine City of Hope Duarte CA USA

**Keywords:** Lymphoma, Haematopoietic cell transplantation, Sarcopenia, Obesity, Mortality, complications

## Abstract

**Background:**

The number of patients undergoing autologous haematopoietic cell transplant (HCT) is growing, but little is known about the factors that predict adverse outcomes. Low muscle mass and obesity are associated with disability and premature mortality in individuals with non‐malignant diseases and may predict outcomes after autologous HCT.

**Methods:**

This was a retrospective cohort study of 320 patients who underwent autologous HCT for Hodgkin or non‐Hodgkin lymphoma between 2009 and 2014. Sarcopenia {skeletal muscle index male: <43 cm/m^2^ [body mass index (BMI) < 25 kg/m^2^] or < 53 cm/m^2^ [BMI ≥ 25 kg/m^2^] and female: <41 cm/m^2^ [regardless of BMI]) and obesity [total abdominal adiposity ≥450.0 cm^2^ (male), ≥396.4 cm^2^ (female)] were assessed from single‐slice abdominal pre‐HCT computed tomography images. Length of hospital stay, first unplanned intensive care unit admission, and 30‐day unplanned readmission were evaluated based on body composition using multivariable regression analysis, and mortality was evaluated with Kaplan–Meier analysis and Gray's test.

**Results:**

Median age at HCT was 53.3 years (range, 18.5 to 78.1 years); 26.3% were sarcopenic and an additional 7.8% were sarcopenic obese pre‐HCT. Sarcopenic obesity was associated with increased risk of prolonged hospitalization [odds ratio (OR) = 3.6, 95% confidence interval (CI) 1.3–9.8], intensive care unit admission (OR = 4.7, 95% CI 1.5–16.1), and unplanned readmission after HCT (OR = 13.6, 95% CI 2.5–62.8). Patients who were sarcopenic obese also had the highest mortality risk at 1 year [hazard ratio (HR): 3.9, 95% CI 1.1–11.0] and 5 years (HR: 2.5, 95% CI 1.1–5.5), compared with patients with normal body composition. Sarcopenia alone, but not obesity alone, was associated with an increased risk of these outcomes, albeit with a lower magnitude of risk than in patients who were sarcopenic obese.

**Conclusions:**

Sarcopenic obesity was an important predictor of outcomes in patients undergoing autologous HCT. These findings could inform targeted prevention strategies in patients at highest risk of complications after HCT.

## Introduction

Autologous haematopoietic cell transplantation (HCT) is an established treatment approach in patients with lymphoma, either as a frontline therapy or as more commonly, in the relapsed/refractory setting.^1^, ^2^, ^3^ Advances in HCT strategy and supportive care have steadily increased the number of HCTs performed each year, and it is estimated there are >12,000 autologous HCTs performed in the USA annually.^4^, ^5^ In these patients, the treatment efficacy of autologous HCT must be balanced by the risk of adverse outcomes, which can diminish quality of life and increase mortality risk.^6^, ^7^, ^8^ Therefore, it is imperative to identify patient subgroups who might be at increased risk of adverse outcomes after HCT and incorporate that information into risk‐reduction strategies that maximize the benefits of treatment.

Conventional methods for predicting adverse outcomes from HCT include the Karnofsky Performance Score (KPS) and the HCT‐comorbidity index (HCT‐CI).^9^, ^10^, ^11^ However, the effectiveness of these measures is limited by high inter‐user variability and low predictive power for outcomes other than survival.^12^ Advances in software technology have made it possible to use computed tomography (CT) images to assess body composition,^13^, ^14^ allowing researchers to determine the extent to which abnormalities in body composition, such as obesity and sarcopenia, might predict HCT outcomes.

Sarcopenia is the progressive loss of muscle mass and muscle function caused by aging, inactivity, and disease, and it is associated with physical disability, frailty, and premature mortality; outcomes are especially poor for patients with both sarcopenia and obesity.^15^, ^16^ In a retrospective analysis of CT images collected pre‐HCT from adult leukaemia patients treated with allogeneic HCT, we identified sarcopenia, which was present in 33.7% of patients, as an independent risk factor for longer hospitalization and mortality.^17^ However, it was not known whether other measures of body composition, such as obesity, can influence health outcomes, especially among autologous HCT patients who represent the largest number of HCT patients. The current study builds on prior work^17^ by examining the association of both obesity and sarcopenia with a broader range of health outcomes [e.g. requirement for intensive care unit (ICU), 30‐day readmission rate, prolonged length of hospitalization, and survival] among autologous HCT patients. Further, we examined the trajectory of change in muscle mass and adiposity over time, by comparing radiographic studies obtained prior to and after HCT.

## Methods

### Population cohort and data definitions

This was a retrospective cohort study of patients who underwent autologous HCT for non‐Hodgkin lymphoma (NHL) or Hodgkin lymphoma (HL) as adults (≥18 years old) at City of Hope (COH) between 1 January 2009 and 31 December 2014. Patients were identified from the long‐term follow‐up after HCT research programme at COH, which ensures the active and comprehensive follow‐up of all patients who have undergone HCT at COH since 1976. Patients are consented *prior to* HCT, and follow‐up is performed by a dedicated research team throughout the life of the survivor. A data collection form is completed by research staff, capturing demographics, cancer diagnosis, pre‐HCT treatment information (e.g. radiation and chemotherapy), and HCT‐related exposures (e.g. conditioning intensity). Importantly, this protocol uses a number of strategies (questionnaire, medical record abstraction, and national registries) to collect information on health‐related outcomes after HCT. Complications are coded using a standardized set of definitions for each outcome, with appropriate source documentation (e.g. pathology reports and imaging studies) available for confirmation of outcomes. This information is entered into an electronic database that can be queried for research studies, allowing us to accurately describe the epidemiology and risk factors {demographics, pre‐HCT, HCT‐related treatment exposures [e.g. conditioning therapy such as carmustine, etoposide, cytarabine and melphalan (BEAM)], and comorbidities at the time of HCT} for *de novo* health‐related complications after HCT. The COH long‐term follow‐up after HCT research programme has been approved by the COH Institutional Review Board (IRB# 00029), and data abstraction and associated procedures are performed in accordance with the Declaration of Helsinki. For the current study, medical records were abstracted for patient demographics (age at HCT, sex, and race/ethnicity), diagnosis, remission status at HCT [complete radiographic remission (CR) or not in CR], variables necessary to calculate the HCT‐CI using an established web‐based calculator (www.hctci.org),^17^, ^18^ KPS, conditioning therapy, body mass index (BMI) at HCT, unplanned ICU admission during HCT (first admission occurring between the start of conditioning therapy and HCT hospital discharge), hospital length of stay (LOS), and re‐hospitalization within 30 days of discharge [planned, unplanned (e.g. dehydration, fever in an immunocompromised host, organ dysfunction)]. Risk factors such as history of tobacco and alcohol use prior to HCT were not abstracted because they were not consistently documented in patient medical records. Information on vital status and cause of death was obtained from the National Death Index and COH medical records. High HCT‐CI was defined as having a pre‐HCT comorbidity index ≥3, a threshold that has been consistently associated with adverse health outcomes after HCT.^12^, ^18^ Good performance status was defined as KPS >80. Hospital LOS was determined from the start of conditioning therapy to discharge or death, and prolonged LOS was defined as ≥24 days (upper tertile for the cohort).

Of the 440 patients who underwent autologous HCT for HL or NHL as adults between 2009 and 2014, 109 were excluded from the current study because of a lack of available CT images for review, and an additional 11 were excluded because the abdominal CT images were >90 days before HCT (*N* = 5) or because the images were of poor quality (*N* = 6). With the exception of race/ethnicity, there were no statistically significant differences in patient and treatment‐related characteristics between those included in the study vs. those who were not (see Supporting Information, *Table* S1)

### Computed tomography image analysis

**Figure 1:**
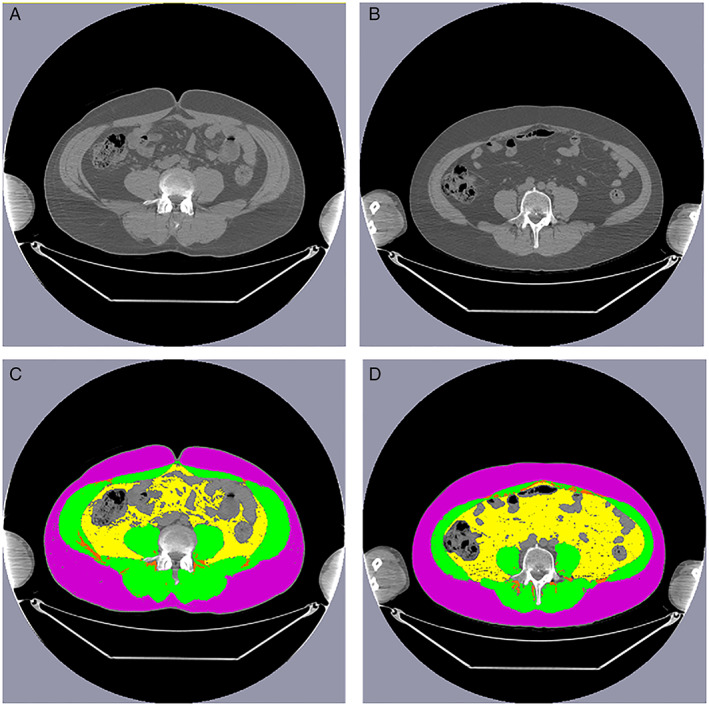
Axial computed tomography images of the third lumbar vertebra region without (top) and with (bottom) annotation showing skeletal muscle (green), subcutaneous adipose tissue (purple), visceral adipose tissue (yellow), intra‐muscular and inter‐muscular adipose tissue (orange) for patients (A) without and (B) with sarcopenic obesity. Characteristics of both patients: male, 50 years old at haematopoietic cell transplant, diagnosis: non‐Hodgkin lymphoma, BEAM conditioning, low (<3) haematopoietic cell transplant‐comorbidity index. Patient on the left (A): body mass index 29.3 kg/m^2^, skeletal muscle index 63.9 cm/m^2^, subcutaneous adipose tissue area 271.2 cm^2^, visceral adipose tissue area 53.0 cm^2^, intra‐muscular and inter‐muscular adipose tissue area 9.2 cm^2^; Patient on the right (B): body mass index 27.5 kg/m^2^, skeletal muscle index 46.7 cm/m^2^, subcutaneous adipose tissue area 267.9 cm^2^, visceral adipose tissue area 185.7 cm^2^, intra‐muscular and inter‐muscular adipose tissue area 7.8 cm^2^.

The quantity of muscle and adipose tissue was measured on CT images, which were collected either alone or as part of positron emission tomography imaging (GE Discovery STE PET/CT; GE Healthcare; Chicago, IL)^19^ in 11.2% and 88.8% of patients, respectively. Pre‐HCT scans were limited to those within 90 days of stem cell infusion [median 28 days (range, 0 to 90 days)], and post‐HCT scans were limited to those <180 days [median 57 days (range, 22 to 162 days)] after infusion. Two trained (A.I. and K.M.) researchers quantified the cross‐sectional area of muscle and adipose tissue (cm^2^) at the third lumbar vertebra (L3); an inter‐observer coefficient of <2% was required for 32 patients (10% of the cohort) selected at random, a threshold that is consistent with similar reports in the literature.^13^, ^14^, ^17^ Single‐slice abdominal cross‐sectional area at L3 is highly (>0.9) correlated with whole‐body volumes of muscle and adipose tissue.^20^, ^21^, ^22^, ^23^, ^24^, ^25^ Tissue‐specific Hounsfield unit (HU) ranges were used to discriminate between muscle and adipose tissue, using SliceOmatic Software (Region Growing Module; Software v.5.0 TomoVision, Montreal, Quebec, Canada; *Figure* 1)^14^, ^24^, ^26^, ^27^; intra‐class coefficients between the SliceOmatic software and other software are highly similar (0.979 to 1.000, *P* < 0.001),^13^, ^14^ with excellent intra‐ observer and inter‐observer agreement for muscle and adiposity measurements (≥0.98).^13^, ^14^, ^17^

The following muscles were included in the muscle area measurements (HU range −29 to 150): psoas, paraspinal (quadratus lumborum and erector spinae), transversus abdominis, external and internal obliques, and rectus abdominis. The skeletal muscle index (SMI) was defined as the muscle area at L3 divided by height.^22^, ^23^, ^24^, ^25^ We used an a priori definition of sarcopenia,^9^, ^18^ categorized by sex and BMI‐specific cutoffs for abnormal SMI [male: <43 cm/m^2^ (BMI < 25 kg/m^2^) or <53 cm/m^2^ (BMI ≥ 25 kg/m^2^) and female: <41 cm/m^2^ (regardless of BMI)]. Measures of adiposity included the following: visceral (intra‐abdominal) adipose tissue (VAT; HU range −150 to −50), subcutaneous adipose tissue (SAT; HU range −190 to −30), intramuscular adipose tissue (IMAT, HU range −190 to −30). Total adiposity (TAT) was the sum of VAT, SAT, and IMAT. Because of a lack of well‐established definitions for these adiposity measures, we used sex‐specific upper tertiles as the cutoff for abnormal VAT [≥170.0 cm^2^ (male), ≥100.0 cm^2^ (female)], SAT [≥265.0 cm^2^ (male), ≥283.0 cm^2^ (female)], IMAT [>15.0 cm^2^ (male), >13.4 cm^2^ (female)], TAT [≥450.0 cm^2^ (male), ≥396.4 cm^2^ (female)]. Obesity was defined as abnormal TAT, and sarcopenic obesity was defined as having both an abnormal SMI and an abnormal TAT. Separate exploratory analyses were performed using the established BMI‐derived definition (≥30 kg/m^2^) of obesity (*Table* S2).

### Statistical analysis

Univariate analyses (χ^2^ test for categorical, *t*‐test or analysis of variance for parametric continuous variables, Kruskal–Wallis for non‐parametric) were performed to compare baseline demographic, clinical, treatment‐related factors, and body composition measures between male and female patients. Additionally, we compared LOS, ICU admission, 30‐day unplanned readmission rate by body composition phenotype, categorized as (i) normal body composition (not sarcopenic and not obese), (ii) obese (obese and not sarcopenic), (iii) sarcopenic (not obese and sarcopenic), (iv) sarcopenic obese (obese and sarcopenic). We used paired *t*‐tests to compare changes in muscle and adiposity measures between pre‐HCT and post‐HCT scans. The Kaplan–Meier method was used to examine the effect of body composition measures on overall survival; log‐rank tests were used to compare survival rates for the overall cohort and by sex (*Figures* S1 and S2). We also examined the cumulative incidence of cause‐specific (relapse‐related and non‐relapse‐related) mortality for the overall cohort and by sex, taking into consideration competing risk of death for right‐censored data; we used Gray's test^28^ to compare the cumulative incidence curves (*Figure* S3). Patients alive at last contact were right‐censored at 5 years.^28^

Multivariable regression models were created to examine the impact of abnormal body composition (categorical) on select health outcomes {logistic regression [adjusted odds ratio (OR)]: prolonged LOS [≥24 days, <24 days], ICU admission [yes, no], 30‐day unplanned readmission [yes, no]; Cox‐proportional hazard models [hazard ratio (HR)]: 1‐ and 5‐year survival}, adjusting for a priori‐determined variables. These variable included: age at HCT (continuous), sex, HCT‐CI (<3, ≥3), KPS (continuous), lymphoma diagnosis (Hodgkin, diffuse large b‐cell lymphoma, mantle cell, follicular, T‐cell, other), and remission status at HCT (CR or not in CR); as a result of high collinearity between conditioning therapy and diagnosis, conditioning therapy was not included in the final models. A two‐sided *P* value <0.05 was considered statistically significant.

## Results

### Patient characteristics

The median age at HCT was 50.8 years (range 18.5 to 78.1 years), and the majority of patients were male (61.9%), non‐Hispanic white (55.6%), had a diagnosis of diffuse large b‐cell lymphoma (56.4%), received BEAM conditioning (57.1%), were in complete remission at HCT (56.3%), had a good KPS (>80, 72.2%), and had a low HCT‐CI score [(0–2) 83.8%] at HCT (*Table* 1). With the exception of the NHL subtype, there were no statistically significant differences in the demographic, clinical, and treatment characteristics by sex. As expected, there were significant differences in the body composition measures between male and female patients, with male patients having increased height, weight, BMI, SMI, VAT, SAT, and TAT pre‐HCT, compared with female patients. Interestingly, there was modest correlation [R range: 0.232 (weak) to 0.671 (moderate)] between imaging parameters and conventional measures such as body weight and BMI irrespective of sex (*Table* S3). Overall, 84 patients (26.3% of the cohort) were sarcopenic non‐obese and an additional 25 (7.8%) were sarcopenic obese prior to HCT.

**Table 1:** Demographic and clinical characteristics of haematopoietic cell transplantation patients

Characteristics	Overall cohort (*N* = 320)	Male (*n* = 198)	Female (*n* = 122)	*P* value
Age at HCT (years)				
Median (range)	53.3 (18.5–78.1)	53.5 (18.5–78.1)	52.5 (19.7–72.0)	
Mean (SD)	50.8 (13.7)	51.6 (13.2)	49.5 (14.3)	0.172
Race/Ethnicity, No. (%)				
Non‐Hispanic white	178 (55.6)	107 (54.0)	71 (58.2)	
Hispanic	83 (25.9)	56 (28.3)	27 (22.1)	
Asian	39 (12.2)	12 (11.6)	16 (13.1)	
Other	20 (6.3)	12 (6.1)	8 (6.6)	0.681
Diagnosis, No. (%)				
Hodgkin lymphoma	84 (26.2)	48 (24.2)	36 (29.5)	
Non‐Hodgkin lymphoma	236 (73.8)	150 (75.8)	86 (70.5)	0.298
DLBCL	133 (56.4)	74 (49.3)	59 (68.6)	
Mantle cell	50 (21.2)	43 (28.7)	7 (8.1)	
Follicular	24 (10.2)	14 (9.3)	10 (11.6)	
T‐cell	21 (8.9)	13 (8.7)	8 (9.3)	
Other	8 (3.4)	6 (4.0)	2 (2.3)	0.004^a^
Conditioning, No. (%)				
BEAM	183 (57.1)	114 (57.6)	69 (56.6)	
CBV	126 (39.4)	78 (39.4)	48 (39.3)	
Other	11 (3.4)	6 (3.0)	5 (4.1)	0.876
Remission status at HCT, No. (%)				
Complete remission	180 (56.3)	109 (55.1)	71 (58.2)	
Not in complete remission	140 (43.8)	89 (44.9)	51 (41.8)	0.582
Karnofsky performance score, No. (%)				
>80	231 (72.2)	143 (72.2)	88 (72.1)	
≤80	86 (26.9)	54 (27.3)	32 (26.2)	
Unknown	3 (0.9)	1 (0.5)	2 (1.6)	0.587
HCT‐comorbidity index, No. (%)				
0–2	268 (83.8)	167 (84.3)	101 (82.8)	
≥3	52 (16.2)	31 (15.7)	21 (17.2)	0.714
Body mass index				
Mean, kg/m^2^ (SD)	28.3 (5.7)	29.2 (5.4)	26.9 (6.0)	<0.001
<25 kg/m^2^	92 (28.8)	38 (19.2)	54 (44.3)	
25–29.9 kg/m^2^	125 (39.7)	88 (44.4)	37 (30.3)	
≥30 kg/m^2^	103 (32.2)	72 (36.4)	31 (25.4)	<0.001
Height				
Mean, cm (SD)	170.4 (9.0)	174.7 (6.9)	163.3 (7.5)	<0.001
Weight				
Mean, kg (SD)	82.7 (19.7)	89.4 (18.5)	71.1 (16.3)	<0.001
Skeletal muscle index				
Mean, cm/m^2^ (SD)	50.8 (10.3)	56.1 (7.9)	42.2 (7.7)	<0.001
Visceral adipose tissue area				
Mean, cm^2^ (SD)	122.4 (81.5)	143.7 (87.0)	87.8 (56.9)	<0.001
Subcutaneous adipose tissue area				
Mean, cm^2^ (SD)	255.9 (118.1)	250.8 (114.8)	264.1 (123.1)	0.329
Intramuscular adipose tissue area				
Mean, cm^2^ (SD)	12.0 (7.5)	11.7 (7.1)	12.4 (8.0)	0.430
Total abdominal tissue area				
Mean, cm^2^ (SD)	390.8 (169.0)	406.6 (173.8)	365.2 (158.4)	0.033

BEAM, carmustine (BCNU), etoposide, cytarabine (ARA‐C), melphalan; CBV, cyclophosphamide, BCNU, etoposide (VP‐16); DLBCL, diffuse large B‐cell lymphoma; HCT, haematopoietic cell transplantation; No., number; SD, standard deviation.

a Subset analysis within non‐Hodgkin lymphoma.

### Health outcomes by pre‐haematopoietic cell transplantation body composition measures

#### Length of hospitalization

Compared with patients with normal body composition, patients who were sarcopenic obese had the longest LOS (mean 28.6 days vs. 23.4 days), followed by those who were only sarcopenic (mean 24.6 days); *P* = 0.003; *Table* 2. The odds of having a prolonged LOS was 2.0‐fold (95% CI 1.2–3.5) higher among patients who were sarcopenic (reference: normal body composition), and the odds were highest for patients with sarcopenic obesity (OR: 3.6, 95% CI 1.3–9.8; *Table* 2).

**Table 2:** Pre‐haematopoietic cell transplantation body composition measures and short‐term health outcomes. *P* values <0.05 are in bold font

	Mean LOS, days (SD)	*P* value	Prolonged LOS,^a^ *N* (%)	*P* value	Unadjusted OR (95%CI)	Adjusted^b^ OR (95%CI)	ICU admission *N*, (%)	P value	Unadjusted OR (95%CI)	Adjusted^b^ OR (95%CI)	30 days readmit *N*, (%)	*P* value	Unadjusted OR (95%CI)	Adjusted^b^ OR (95%CI)
Body mass index														
<25 kg/m^2^ (*N* = 90)	23.5 (3.5)	0.112	31 (34.4)	0.949	1.0	1.0	13 (14.4)	0.933	1.0	1.0	4 (4.4)	0.582	1.0	1.0
25–29.9 kg/m^2^ (*N* = 127)	23.9 (6.3)		43 (33.9)		1.0 (0.6–1.7)	0.8 (0.4–1.5)	17 (13.4)		0.9 (0.4–2.0)	0.9 (0.4–2.1)	10 (7.9)		1.8 (0.5–6.1)	2.0 (0.5–7.3)
≥30 kg/m^2^ (*N* = 103)	25.4 (8.7)		37 (35.9)		1.1 (0.6–1.9)	1.0 (0.5–2.0)	13 (12.6)		0.9 (0.4–2.0)	0.7 (0.3–1.7)	6 (5.9)		1.4 (0.4–5.0)	1.0 (0.2–3.9)
Sarcopenia														
No (*N* = 211)	23.6 (6.2)	**0.013**	58 (27.5)	**<0.001**	1.0	1.0	18 (8.5)	**<0.001**	1.0	1.0	8 (3.8)	**0.011**	1.0	1.0
Yes (*N* = 109)	25.5 (7.2)		53 (48.6)		2.5 (1.5–4.0)	2.0 (1.2–3.5)	25 (22.9)		3.2 (1.7–6.2)	3.7 (1.8–7.8)	12 (11.1)		3.2 (1.3–8.0)	6.4 (2.1–19.5)
Visceral adiposity														
No (*N* = 215)	23.8 (6.7)	0.140	66 (30.7)	**0.032**	1.0	1.0	26 (12.1)	0.313	1.0	1.0	17 (7.9)	0.081	1.0	1.0
Yes (*N* = 105)	25.0 (6.6)		45 (42.9)		1.7 (1.0–2.8)	1.1 (0.6–1.8)	17 (16.2)		1.4 (0.7–2.7)	1.0 (0.5–2.1)	3 (2.9)		0.3 (0.1–1.2)	0.4 (0.1–1.4)
Subcutaneous adiposity														
No (*N* = 214)	24.1 (6.3)	0.566	74 (34.6)	0.954	1.0	1.0	27 (12.6)	0.541	1.0	1.0	13 (6.1)	0.847	1.0	1.0
Yes (*N* = 106)	24.5 (7.2)		37 (34.9)		1.0 (0.6–1.7)	1.2 (0.7–2.1)	16 (15.1)		1.2 (0.6–2.4)	1.0 (0.5–2.0)	7 (6.7)		1.1 (0.4–2.8)	1.0 (0.3–2.6)
Intramuscular adiposity														
No (*N* = 213)	23.7 (6.2)	0.072	65 (30.5)	**0.027**	1.0	1.0	28 (13.1)	0.829	1.0	1.0	15 (7.0)	0.213	1.0	1.0
Yes (*N* = 107)	25.2 (7.4)		46 (43.0)		1.7 (1.1–2.8)	1.1 (0.6–1.9)	15 (14.0)		1.1 (0.6–2.1)	0.8 (0.4–1.6)	5 (4.7)		0.5 (0.0–2.3)	0.4 (0.1–1.5)
Total abdominal adiposity														
No (*N* = 214)	23.9 (6.3)	0.185	68 (31.8)	0.120	1.0	1.0	28 (13.1)	0.792	1.00	1.0	14 (6.0)	0.767	1.0	1.0
Yes (*N* = 106)	24.9 (7.3)		43 (40.6)		1.5 (0.9–2.4)	1.4 (0.8–2.5)	15 (14.2)		1.1 (0.6–2.2)	0.9 (0.4–1.8)	6 (5.7)		0.9 (0.3–2.3)	0.8 (0.3–2.4)
Body composition														
Not sarcopenic, Not obese (*N* = 130)	23.4 (7.1)	**0.003**	30 (23.1)	**<0.001**	1.0	1.00	11 (8.5)	**0.002**	1.0	1.0	6 (4.7)	**0.037**	1.0	1.0
Not sarcopenic, obese (*N* = 81)	23.8 (4.6)		28 (34.6)		1.8 (0.9–3.3)	1.8 (1.0–3.6)	7 (8.6)		1.0 (0.4–2.8)	0.8 (0.3–2.2)	2 (2.5)		0.5 (0.1–2.6)	0.5 (0.1–2.6)
Sarcopenic, not obese (*N* = 84)	24.6 (4.7)		38 (45.2)		2.8 (1.5–5.0)	2.6 (1.3–5.3)	17 (20.2)		2.8 (1.2–6.2)	3.0 (1.2–7.5)	8 (9.5)		2.2 (0.7–6.5)	4.0 (1.1–14.2)
Sarcopenic obese (*N* = 25)	28.6 (12.1)		15 (60.0)		5.0 (2.0–12.3)	3.6 (1.3–9.8)	8 (32.0)		5.1 (1.8–14.5)	4.7 (1.5–16.1)	4 (16.7)		4.1 (1.1–15.8)	13.6 (2.5–62.8)

CI, confidence interval; ICU, intensive care unit; LOS, length of hospital stay for HCT; *N*, number; OR, odds ratio; Readmit, readmission

a Defined as ≥24 days (upper tertile for the cohort).

b Multivariable logistic regression; model adjusted for age at HCT, sex, HCT‐specific comorbidity index, Karnofsky performance status, diagnosis, remission status at HCT.

#### Intensive care unit admission

Forty‐three (13.4%) patients required ICU admission during their HCT. Compared with patients with normal body composition, patients who were sarcopenic obese had the highest rate of ICU admission (32.0% vs. 8.5%), followed by those who were only sarcopenic (20.2%); *P* = 0.002; *Table* 2. The odds of requiring ICU admission were 3.7‐fold (95% CI 1.8–7.8) higher among patients who were sarcopenic (reference: normal body composition), and the odds were highest for patients with sarcopenic obesity (OR: 4.7, 95% CI 1.5–16.1; *Table* 2).

#### Unplanned readmission

Twenty (6.25%) patients had an unplanned readmission within 30 days of hospital discharge. Compared with patients with normal body composition, patients who were sarcopenic obese had the highest rate of readmission (16.7% vs. 4.7%; *Table* 2), followed by those who were only sarcopenic (9.5%); *P* = 0.037; *Table* 2. The odds of unplanned readmission were 6.4‐fold (95% CI 2.1–19.5) higher among patients who were sarcopenic (reference: normal body composition), and the odds were highest for patients with sarcopenic obesity (OR: 13.6, 95% CI 2.5–62.8; *Table* 2).

#### Overall survival after haematopoietic cell transplantation

**Figure 2:**
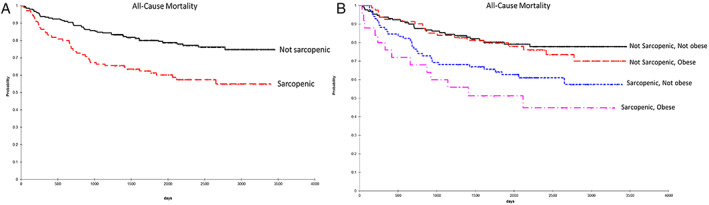
Kaplan–Meier plots of overall survival according to (A) pre‐haematopoietic cell transplant sarcopenia, and (B) the combination of sarcopenia and abdominal obesity.

The overall survival probability of the cohort was 90.3% at 1 year and 73.9% at 5 years after HCT. The 1 and 5 year survival probabilities were significantly worse for patients who were sarcopenic compared with those who were not (1 year: 83.6% vs. 93.4%, *P* = 0.006; 5 years: 61.3% vs. 80.1%, *P* < 0.001; *Table* 3, *Figure* 2A). These group differences persisted, irrespective of cause of death (relapse‐related or non‐relapse‐related); *Figure* S1. Sarcopenia was associated with a 2.5‐fold risk (95% CI 1.0–5.4) of all‐cause mortality at 1 year and 1.8‐fold risk (95% CI 1.1–2.9) of all‐cause mortality at 5 years after HCT (*Table* 3). Notably, patients who were sarcopenic obese had the lowest survival probabilities (1 year: 76.0%; 5 years: 51.33%; *Table* 3, *Figure* 2), and had the highest 1 year (HR: 3.9, 95% CI 1.1–11.0) and 5 year (HR: 2.5, 95% CI 1.1–5.5) mortality risks (*Table* 3).

**Table 3:** Survival probability and risk of one‐ and five‐year all‐cause mortality after haematopoietic cell transplantation

	1‐year all‐cause mortality						5‐year all‐cause mortality					
	Survival probability (%; 95% CI)	*P* value	Unadjusted hazard ratio (95% CI)	*P* value	Adjusted^a^ hazard ratio (95% CI)	*P* value	Survival probability (%; 95% CI)	*P* value	Unadjusted hazard ratio (95% CI)	*P* value	Adjusted^a^ hazard ratio (95% CI)	*P* value
Model 1												
Not sarcopenic	93.4 (89.1–96.0)		1.0		1.0		80.1 (74.0–85.0)		1.0		1.0	
Sarcopenic	83.6 (75.3–89.4)	0.006	2.6 (1.3–5.2)	0.007	2.5 (1.0–5.4)	0.046	61.3 (51.5–69.8)	<0.001	2.2 (1.4–3.4)	<0.001	1.8 (1.1–2.9)	0.022
Model 2												
Not sarcopenic, not obese	94.0 (88.1–96.9)		1.0		1.0		80.3 (72.2–86.3)		1.0		1.0	
Not sarcopenic, obese	92.6 (84.3–96.6)		1.2 (0.4–3.4)	0.751	1.0 (0.2–3.3)	0.985	79.9 (69.2–87.2)		1.0 (0.5–1.9)	0.953	1.0 (0.5–1.9)	0.976
Sarcopenic, not obese	85.9 (76.5–91.7)		2.4 (0.9–5.8)	0.061	2.1 (0.8–5.5)	0.180	64.3 (53.0–73.5)		2.0 (1.5–3.4)	0.014	1.6 (0.9–2.9)	0.118
Sarcopenic, obese	76.0 (54.2–88.4)	0.015	4.4 (1.5–12.7)	0.006	3.9 (1.1–11.0)	0.042	51.3 (30.4–68.9)	<0.001	3.2 (1.6–6.3)	0.001	2.5 (1.1–5.5)	0.021
			*P* trend	0.004	*P* trend	0.040			*P* trend	<0.001	*P* trend	0.018

a Multivariable Cox regression; model adjusted for age at haematopoietic cell transplantation (HCT), sex, HCT‐specific comorbidity index, Karnofsky performance status, diagnosis, and remission status at HCT.

### Impact of changes in body composition

There were 275 patients (85.9% of the cohort) who had both a pre‐HCT and post‐HCT scan available for review; there were no statistically significant clinical or treatment‐related differences between patients with and without post‐HCT scans (*Table* S4). SMI was the only body composition measure that significantly changed between the pre‐HCT and post‐HCT assessments, with a mean change of −1.5 cm^2^ (*P* < 0.001; *Table* S5). There were 30 (10.9%) patients who developed *de novo* sarcopenia after HCT (i.e. had normal SMI pre‐HCT). Of note, there were no statistically significant demographics, clinical, and treatment‐related predictors of *de novo* sarcopenia (*Table* S6). Patients who developed *de novo* sarcopenia had significantly worse 1 and 5 year overall survival probabilities compared with patients with normal muscle mass (1 year: 93.3% vs. 83.3%, *P* < 0.005; 5 years: 79.0% vs. 52.8%, *P* = 0.013). *De novo* sarcopenia was associated with a significantly higher risk of 1 year (HR: 9.4, 95%CI 2.0–46.8) and 5 year mortality (HR: 2.6, 95%CI 1.3–6.2) compared with patients with no sarcopenia.

## Discussion

In this contemporary cohort of patients undergoing autologous HCT for lymphoma, we found a significant association between pre‐HCT sarcopenia and clinically important outcomes such as LOS, ICU admission, and 30‐day hospital readmission risk after HCT. Patients who were sarcopenic obese fared especially worse across all outcomes. There were also clear differences in 1 and 5 year survival rates by pre‐HCT body composition measures, with sarcopenic obese patients having a 51% 5 year survival rate compared with 80% for patients who had normal body composition. After adjusting for well‐established modifiers of overall survival such as disease status at HCT, age, comorbidity burden, and diagnosis, there was a >2‐fold risk of all‐cause mortality in patients who were sarcopenic obese compared with those who were not. Muscle mass was the only body composition measure that significantly changed from pre‐HCT to post‐HCT. Patients who developed *de novo* sarcopenia after HCT had a significantly higher risk of mortality compared with patients who maintained normal muscle mass throughout. Of note, BMI, the most commonly utilized measure of body habitus, was not significantly associated with any of the adverse outcomes examined in our study. The overall findings of this study speak to the importance of careful evaluation of body composition prior to and after HCT and the need to consider innovative interventions to minimize the short‐term and long‐term adverse consequences in high‐risk patients.

Body composition measures for the current study were acquired from archived CT images obtained as part of standard of care, a strategy that has been successfully used in other oncology and non‐oncology populations.^22^, ^23^, ^24^, ^25^ To date, studies examining the impact of body composition in cancer patients have mostly focused on patients with solid cancers,^20^, ^21^, ^22^, ^29^, ^30^ including those with gastrointestinal cancers, because of their close link with undernutrition and availability of abdominal scans. Our findings are in line with some of the findings reported in two previous retrospective cohort studies of patients who underwent autologous HCT for lymphoma.^31^, ^32^ The first^31^ included a limited assessment of the psoas muscle and found an inverse correlation between psoas muscle mass and length of hospitalization and a higher risk of complications during HCT and re‐hospitalization after HCT in male but not female patients. The second^32^ included a mixed cohort of allogeneic and autologous HCT patients and found a higher risk of complications among sarcopenic patients undergoing allogeneic HCT but not autologous HCT. Our study builds on the previous studies by describing the added impact of adiposity on a broader set of clinically relevant short‐term and long‐term HCT outcomes, using sex‐stratified indices to define abnormal measures of body composition. Furthermore, by using a longitudinal study design, we were able to highlight for the first time the important prognostic information that can be obtained from post‐HCT assessments.

Understanding the impact of change in body composition can allow for implementation of appropriate interventions for primary or secondary prevention. For some high‐risk patients, such as those who are sarcopenic obese, clinicians may consider alternatives to autologous HCT, such as long‐term targeted therapy or immunotherapy, taking into account the relative survival benefit offered by autologous HCT.^3^ Other patients may be candidates for prehabilitation,^33^, ^34^, ^35^ initiated in the 2 to 6 weeks prior to HCT when no systemic therapy is given and continued through the HCT hospitalization, as tolerated.^36^ Optimizing conditioning regimen dose based on more novel body size indices such as fat‐free mass and conducting pharmacokinetic studies in sarcopenic obese patients may also identify simple dose adjustments that may improve relapse‐related mortality (*Figure* S1).^37^, ^38^ Finally, given our findings about the clinical relevance of post‐HCT measures, innovative approaches are needed to screen and treat patients shortly after HCT. These include strength training, dietary optimization, or better management of comorbidities (e.g. testosterone or growth hormone replacement), strategies that have been effective for patients with chronic pulmonary disease^39^, ^40^, ^41^ and those affected by human immunodeficiency virus‐related muscle wasting.^41^, ^42^, ^43^

There are some limitations to our study. First, the analysed images were from scans obtained at a range of pre‐HCT time points for purposes other than measurement of body composition, which may have resulted in inter‐patient variability. However, we used a well‐established protocol to perform blinded ascertainment of the body composition measures, minimized inter‐observer variability (coefficient <2%) through strict quality control, relied on standardized and validated cutoffs for abnormal SMI, and implemented sex‐based cutoffs for abnormal adiposity, an important consideration given the sex‐based differences in body composition at baseline; of note, the use of BMI‐based definitions of sarcopenic obesity resulted in similar magnitudes of risk with many of the outcomes examined, albeit with wider confidence intervals because of the smaller number of patients who were categorized as such (*Table* S2). Second, the study population included patients with many lymphoma subtypes who had likely received a variety of frontline and salvage therapies prior to HCT, making it difficult to isolate the impact of the pre‐HCT clinical course on post‐HCT outcomes. However, our multivariable analyses adjusted for lymphoma subtypes as well as prognostic variables such as remission status and comorbidity burden at HCT.^44^, ^45^ Third, it is not possible to separate the impact of sarcopenia caused by biological aging from the sarcopenia caused by ongoing malignant disease, making mechanistic assessment challenging. Additional limitations include lack of information on pre‐HCT modifiers (e.g. tobacco and alcohol use) of health outcomes after HCT and relatively small number of events for certain outcomes such as 30‐day readmission, which may have contributed to unstable risk estimates. Studies are needed to independently validate our findings (especially as it relates to the internally derived cutoffs for abdominal adiposity), and to examine the relative contribution of other functional measures (e.g. grip strength and walking speed) and blood biomarkers (e.g. inflammatory or aging‐related biomarkers) that can be obtained as part of routine pre‐HCT assessment, as well as the diagnostic accuracy of other imaging platforms (e.g. ultrasound‐based and bioelectrical impedance) that may be available to evaluate body composition prior to HCT.

In conclusion, this is the largest study to comprehensively examine the association between pre‐HCT and post‐HCT measures of body composition and outcomes after autologous HCT. We found a one‐time pre‐HCT measure of sarcopenia, and adiposity was a significant and independent predictor of mortality at 1 and 5 years after HCT, and the association with sarcopenia persisted after HCT as well. Sarcopenic obesity not only limited the duration of survival but also decreased the quality of survival, as patients with sarcopenic obesity were more likely to experience prolonged hospital LOS, ICU admission, and unplanned re‐hospitalization. The information obtained from this study may help clinicians develop risk reduction strategies, such as evaluating alternatives to HCT in patients at highest risk, or better management of comorbid health conditions, such as dietary optimization, increasing physical activity, and strength training, during and after autologous HCT. The growing number of patients undergoing HCT worldwide makes the development of personalized approaches to transplantation imperative, to safeguard the well‐being of patients well‐beyond the immediate post‐HCT period.

## Author contributions

S.H.A designed the research, collected and assembled the data, analysed and interpreted the data, and wrote the paper. C.L.S. and F.L.W. analysed and interpreted the data and contributed to the writing of the paper. A.I., J.B.T, K.M., A.H., J.M.Z., and T.J.S. collected and assembled the data and contributed to the writing of the paper. J.M.S., S.M.M., S.M., L.W.J., S.J.F., and R.N., analysed and interpreted the data and contributed to the writing of the paper.

## Conflict of interest

S.H.A. declares he has no disclosures, A.I. declares he has no disclosures, J.B.T. declares she has no disclosures, K.M. declares she has no disclosures, A.H. declares he has no disclosures, J.S.M. declares she has no disclosures, J.M.Z declares she has no disclosures, S.M.‐M. declares she has no disclosures, S.M. declares she has no disclosures, T.J.S. declares he has no disclosures, J.M.S. declares she has no disclosures, L.W.J. owns stock in Pacylex Inc., C.‐L.S. declares she has no disclosures, S.J.F. declares he has no disclosures, F.L. W. declares she has no disclosures, R.N. declares he has no disclosures.

## Supporting information


**Table S1.** Patients who underwent a first autologous HCT for lymphoma as adults between 2009 and 2014, including those who were ineligible for the current study.
**Table S2.** Exploratory analyses using BMI‐derived definition (≥30 Kg/m2) of obesity.
**Figure S1** Kaplan‐Meier plots of overall survival in males according to (A) pre‐HCT sarcopenia, and (B) the combination of sarcopenia and abdominal obesity.
**Figure S2** Kaplan‐Meier plots of overall survival in females according to (A) pre‐HCT sarcopenia, and (B) the combination of sarcopenia and abdominal obesity.
**Figure S3.** Cumulative incidence of non‐relapse mortality and relapse‐related mortality according to pre‐HCT sarcopenia status for the overall cohort (A), males (B), females (C)
**Table S3.** Correlation between imaging parameters and conventional measures such as body weight and body mass index for the overall cohort and by sex
**Table S4.** Patient characteristics: individuals with and without post‐HCT scans
**Table S5.** Pairwise t‐test comparison of pre‐ and post‐HCT measures
**Table S6.** Multivariable regression analysis, predictors of de novo sarcopenia after HCT
