# Supplementary material for: Abnormal body composition is a predictor of adverse outcomes after autologous haematopoietic cell transplantation
Source: J Cachexia Sarcopenia Muscle. 2020 Mar 25;11(4):962–72. doi: 10.1002/jcsm.12570 (PMC7432567; doi:10.1002/jcsm.12570)
Supplement: Supplementary file 1 — Table S1. Patients who underwent a first autologous HCT for lymphoma as adults between 2009 and 2014, including those who were ineligible for the current study. Table S2. Exploratory analyses using BMI‐derived definition (≥30 Kg/m2) of obesity. Figure S1 Kaplan‐Meier plots of overall survival in males according to (A) pre‐HCT sarcopenia, and (B) the combination of sarcopenia and abdominal obesity. Figure S2 Kaplan‐Meier plots of overall survival in females according to (A) pre‐HCT sarcopenia, and (B) the combination of sarcopenia and abdominal obesity. Figure S3. Cumulative incidence of non‐relapse mortality and relapse‐related mortality according to pre‐HCT sarcopenia status for the overall cohort (A), males (B), females (C) Table S3. Correlation between imaging parameters and conventional measures such as body weight and body mass index for the overall cohort and by sex Table S4. Patient characteristics: individuals with and without post‐HCT scans Table S5. Pairwise t‐test comparison of pre‐ and post‐HCT measures Table S6. Multivariable regression analysis, predictors of de novo sarcopenia after HCT [file JCSM-11-962-s001.docx]

| **Characteristics** | **Overall Cohort**  **(N=440)** | **Eligible**  **(N=320)** | **Not eligible (N=120)** | **P-Value** |
| --- | --- | --- | --- | --- |
| **Age at HCT, years** |  |  |  |  |
| Median (Range) | 53.9 (18.5-78.1) | 53.3 (18.5-78.1) | 56.0 (21.9-75.6) |  |
| Mean (Standard Deviation) | 51.4 (13.7) | 50.8 (13.7) | 53.2 (13.9) | 0.112 |
| **Sex, No. (%)** |  |  |  |  |
| Male | 278 (63.2) | 198 (61.9) | 80 (66.7) |  |
| Female | 162 (36.8) | 122 (38.1) | 40 (33.3) | 0.353 |
| **Race/Ethnicity, No. (%)** |  |  |  |  |
| Non-Hispanic white | 262 (59.5) | 178 (55.6) | 84 (70.0) |  |
| Hispanic | 105 (23.9) | 83 (25.9) | 22 (18.3) |  |
| Asian | 46 (10.5) | 39 (12.2) | 7 (5.8) |  |
| Other | 27 (6.1) | 20 (6.3) | 7 (7.8) | 0.036 |
| **Diagnosis, No. (%)** |  |  |  |  |
| Hodgkin lymphoma | 106 (24.1) | 84 (26.2) | 22 (18.3) |  |
| Non-Hodgkin lymphoma | 334 (75.9) | 236 (73.8) | 98 (81.7) | 0.084 |
| DLBCL | 189 (56.6) | 133 (56.4) | 56 (57.1) |  |
| Mantle cell | 72 (21.6) | 50 (21.2) | 22 (22.4) |  |
| Follicular | 32 (9.6) | 24 (10.2) | 8 (8.2) |  |
| T-cell | 32 (9.6) | 21 (8.9) | 11 (11.2) |  |
| Other | 9 (2.7) | 8 (3.4) | 1 (1.0) | 0.383* |
| **Conditioning, No. (%)** |  |  |  |  |
| BEAM | 258 (58.6) | 182 (56.9) | 76 (63.3) |  |
| CBV | 168 (38.2) | 127 (39.7) | 41 (34.2) |  |
| Other | 14 (3.2) | 11 (3.4) | 3 (2.5) | 0.456 |
| **Remission status at HCT, No. (%)** |  |  |  |  |
| Complete remission | 257 (58.4) | 180 (56.3) | 77 (64.2) |  |
| Not in complete remission | 183 (41.6) | 140 (43.8) | 43 (35.8) | 0.133 |
| **Karnofsky Performance Score, No. (%)** |  |  |  |  |
| >80 | 328 (74.5) | 231 (72.2) | 97 (80.8) |  |
| ≤80 | 108 (24.5) | 86 (26.9) | 22 (18.3) |  |
| Unknown | 4 (0.9) | 3 (0.9) | 1 (0.8) | 0.176 |
| **HCT-Comorbidity Index, No. (%)** |  |  |  |  |
| 0-2 | 365 (83.0) | 268 (83.8) | 97 (80.8) |  |
| ≥3 | 75 (17.0) | 52 (16.2) | 23 (19.2) | 0.469 |
| **Body mass index** |  |  |  |  |
| Mean, kg/m^2^ (SD) | 28.4 (5.7) | 28.3 (5.7) | 28.3 (5.7) | 0.926 |
| <25 kg/m^2^ | 125 (28.4) | 92 (28.8) | 33 (27.5) |  |
| 25-29.9 kg/m^2^ | 179 (40.7) | 125 (39.7) | 54 (45.0) |  |
| ≥30 kg/m^2^ | 136 (30.9) | 103 (32.2) | 33 (27.5) | 0.490 |
| **Height** |  |  |  |  |
| Mean, cm (SD) | 170.4 (9.0) | 170.4 (9.0) | 172.1 (9.7) | 0.108 |
| **Weight** |  |  |  |  |
| Mean, kg (SD) | 82.7 (19.7) | 82.7 (19.7) | 84.2 (19.7) | 0.407 |

**Table S1.** Patients who underwent a first autologous HCT for lymphoma as adults between 2009 and 2014, including those who were ineligible for the current study.

Abbreviations: HCT, hematopoietic cell transplantation; No., number; DLBCL, diffuse large B-cell lymphoma; BEAM, carmustine (BCNU), etoposide, cytarabine (ARA-C), melphalan; CBV, cyclophosphamide, BCNU, etoposide (VP-16);

*Subset analysis within non-Hodgkin lymphoma

**Table S2**. Exploratory analyses using BMI-derived definition (≥30 Kg/m^2^) of obesity.

|  | **Prolonged LOS** | **ICU Admission** | **30d-Readmit** | **1-year all-cause mortality** | **5-year call-cause mortality** |
| --- | --- | --- | --- | --- | --- |
|  | **Adjusted* OR (95%CI)** | **Adjusted* OR (95%CI)** | **Adjusted* OR (95%CI)** | **Adjusted¶ hazard ratio (95% CI)** | **Adjusted¶ hazard ratio (95% CI)** |
| **Obesity** |  |  |  |  |  |
| No | 1.0 | 1.0 | 1.0 | 1.0 | 1.0 |
| Yes | 1.2 (0.7-2.0) | 1.5 (0.7-3.2) | 0.7 (0.2-2.0) | 1.5 (0.7-3.2) | 1.3 (0.8-2.2) |
| **Body composition** |  |  |  |  |  |
| Not sarcopenic,  Not obese (N=120) | 1.0 | 1.0 | 1.0 | 1.0 | 1.0 |
| Not sarcopenic,  obese (N=90) | 1.7  (0.9-3.5) | 1.7  (0.6-4.8) | 1.0  (0.2-4.3) | 1.6  (0.6-4.8) | 1.1  (0.6-2.1) |
| Sarcopenic,  not obese (N=96) | 2.5  (1.3-5.1) | 4.0  (1.6-11.0) | 4.3  (1.2-15.9) | 1.7  (0.6-4.9) | 1.5  (0.8-2.8) |
| Sarcopenic  obese (N=13) | 3.0  (0.9-10.9) | 4.8  (1.1-21.9) | 14.4  (1.8-114.1) | 4.0  (1.1-15.1) | 5.6  (2.6-12.5) |

Abbreviations: LOS, length of hospital stay for HCT; N, number; OR, odds ratio; CI, confidence interval; ICU, intensive care unit; Readmit, readmission

*Multivariable logistic regression; model adjusted for age at HCT, sex, HCT-specific comorbidity index, Karnofsky performance status, diagnosis, remission status at HCT

¶Multivariable Cox regression; model adjusted for age at HCT, sex, HCT-specific comorbidity index, Karnofsky performance status, diagnosis, and remission status at HCT

**
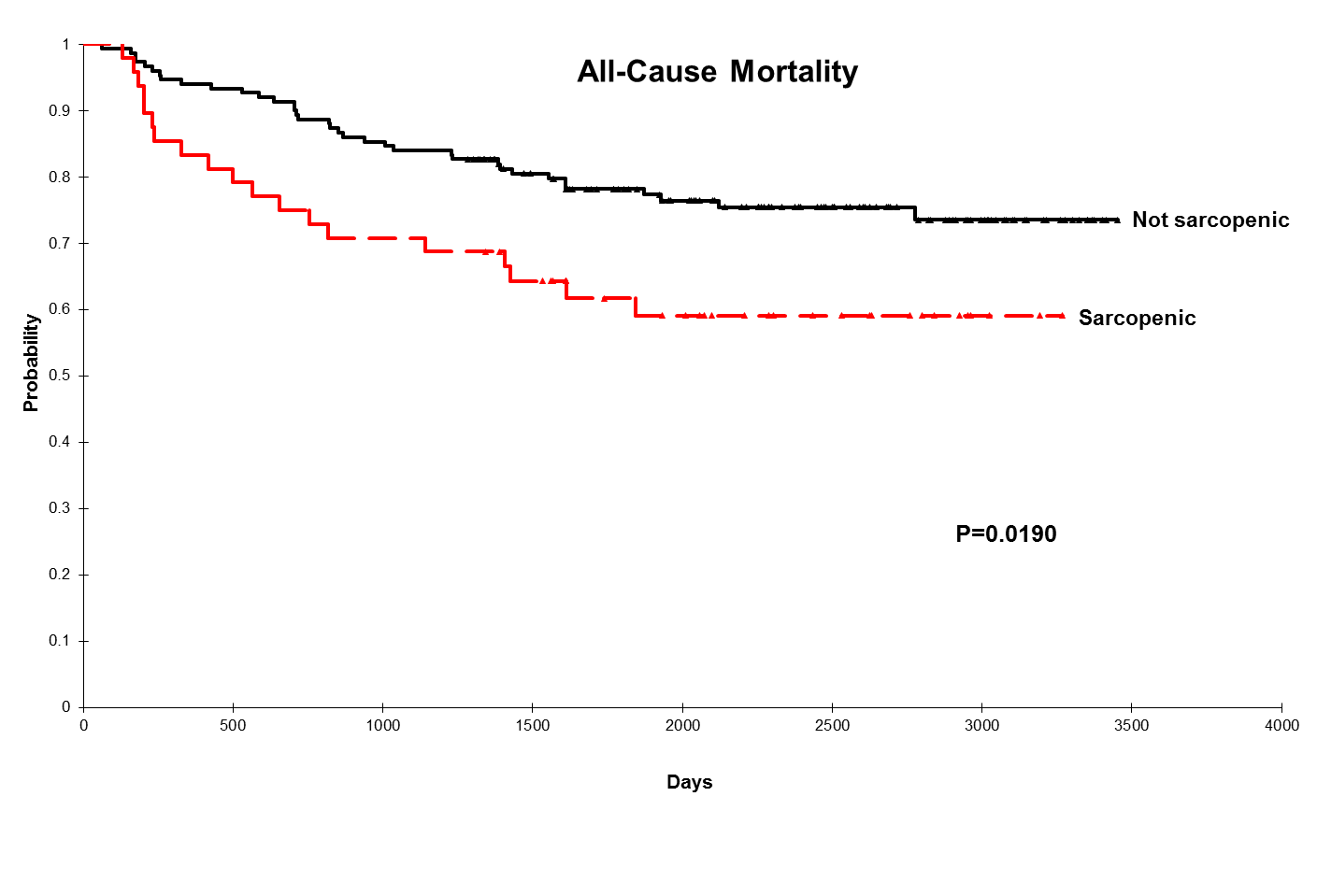
Figure S1** Kaplan-Meier plots of overall survival in males according to **(A)** pre-HCT sarcopenia, and **(B)** the combination of sarcopenia and abdominal obesity.

**
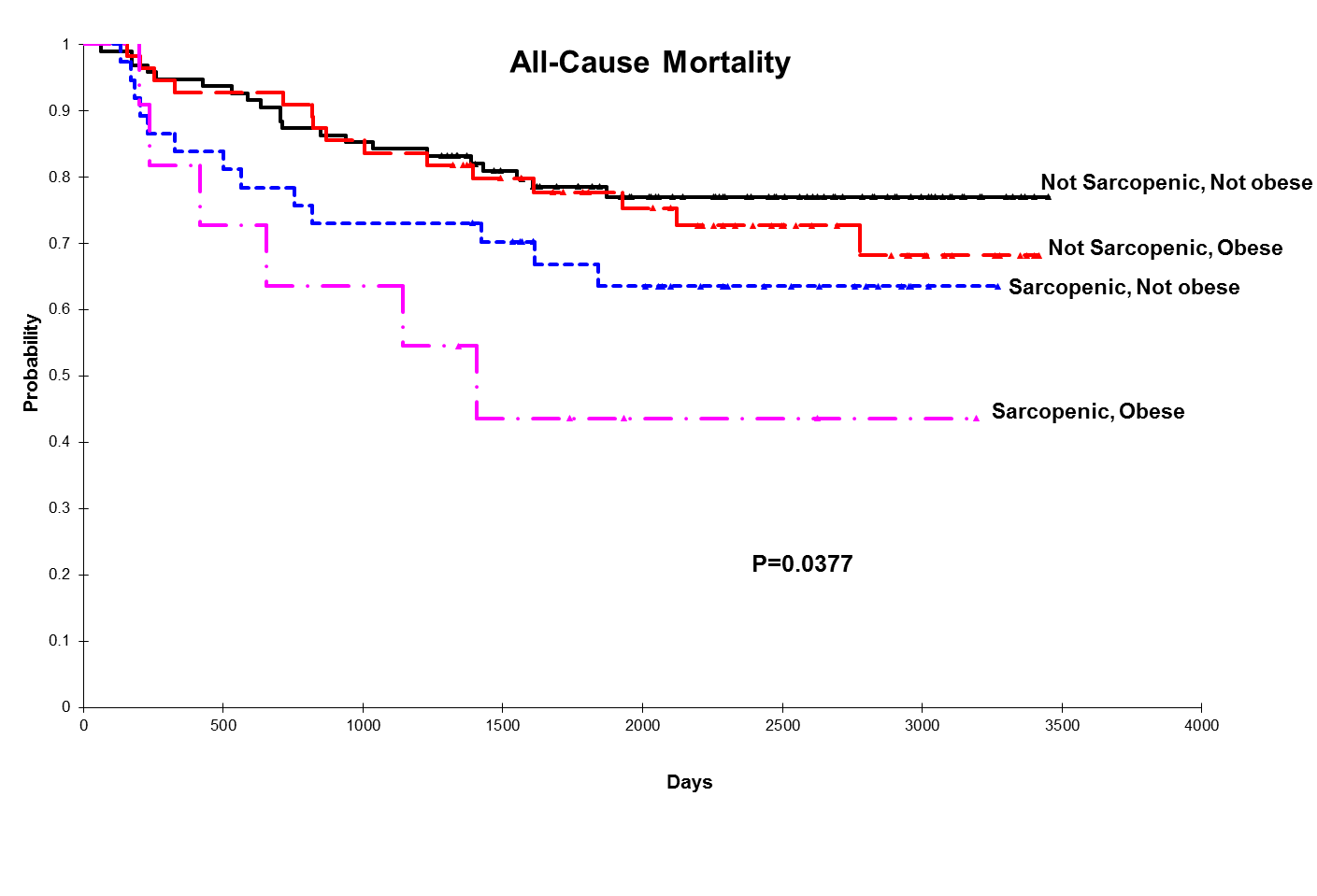
**


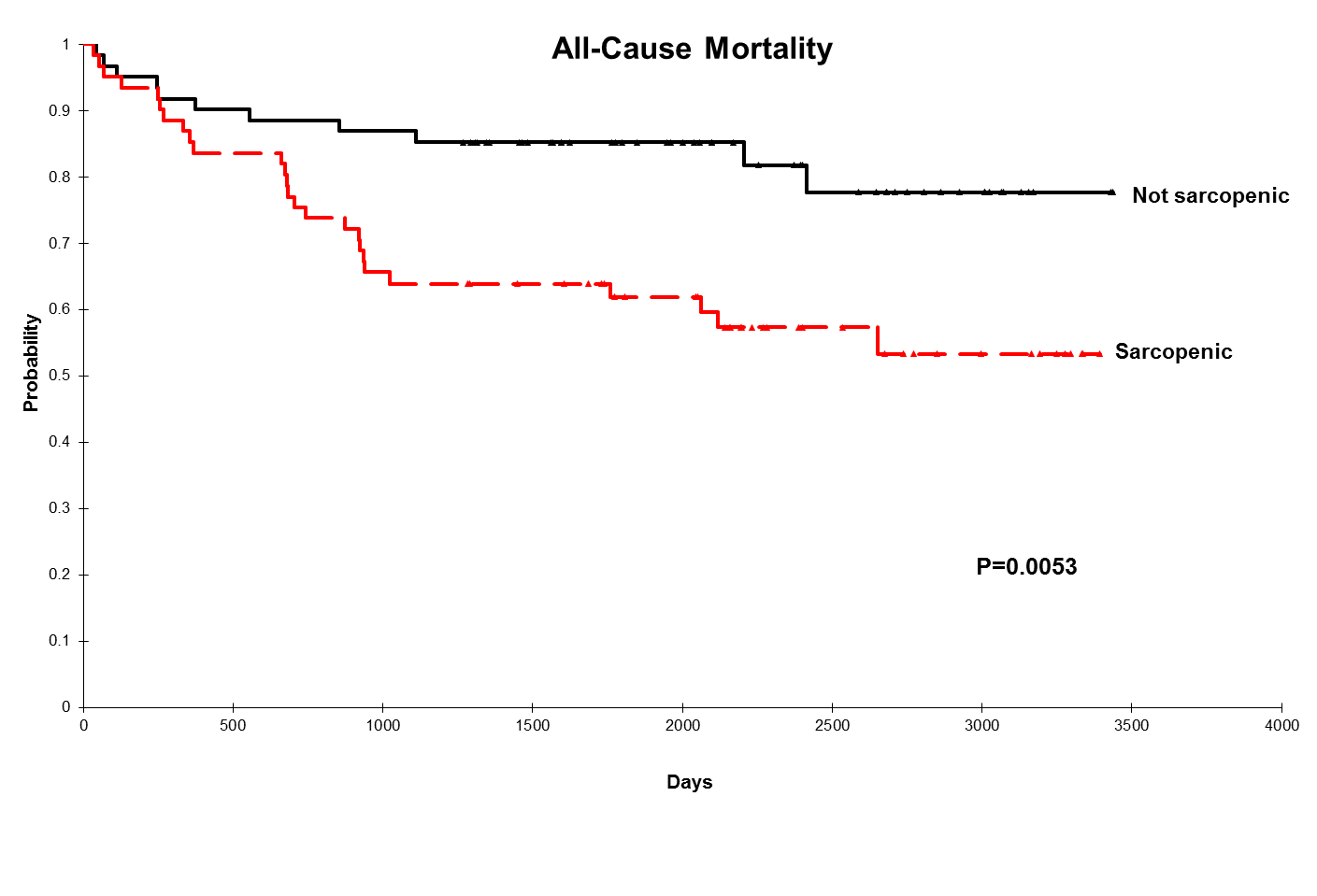
**Figure S2** Kaplan-Meier plots of overall survival in females according to **(A)** pre-HCT sarcopenia, and **(B)** the combination of sarcopenia and abdominal obesity.


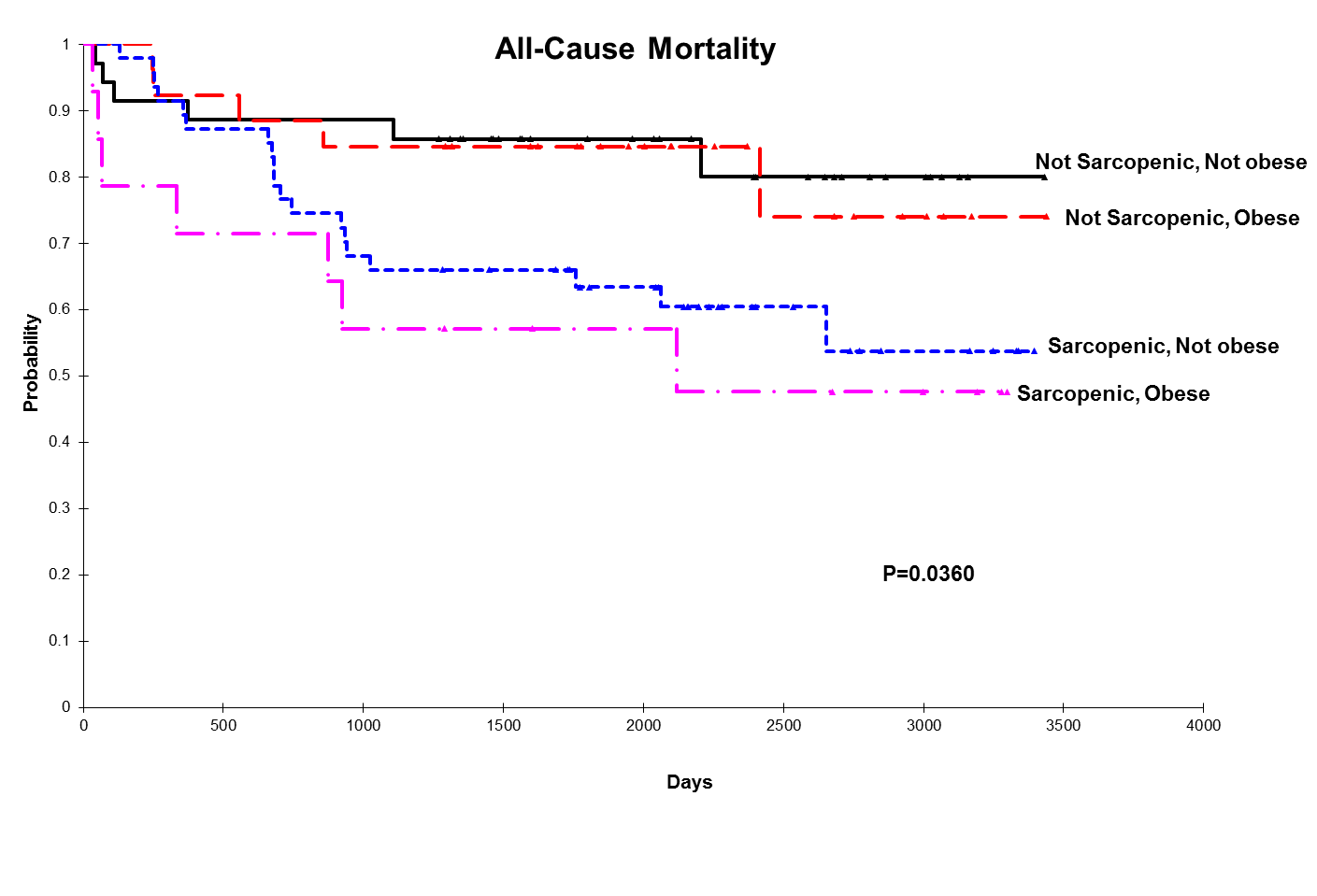


**Figure S3.** Cumulative incidence of non-relapse mortality and relapse-related mortality according to pre-HCT sarcopenia status for the overall cohort (A), males (B), females (C)

A.


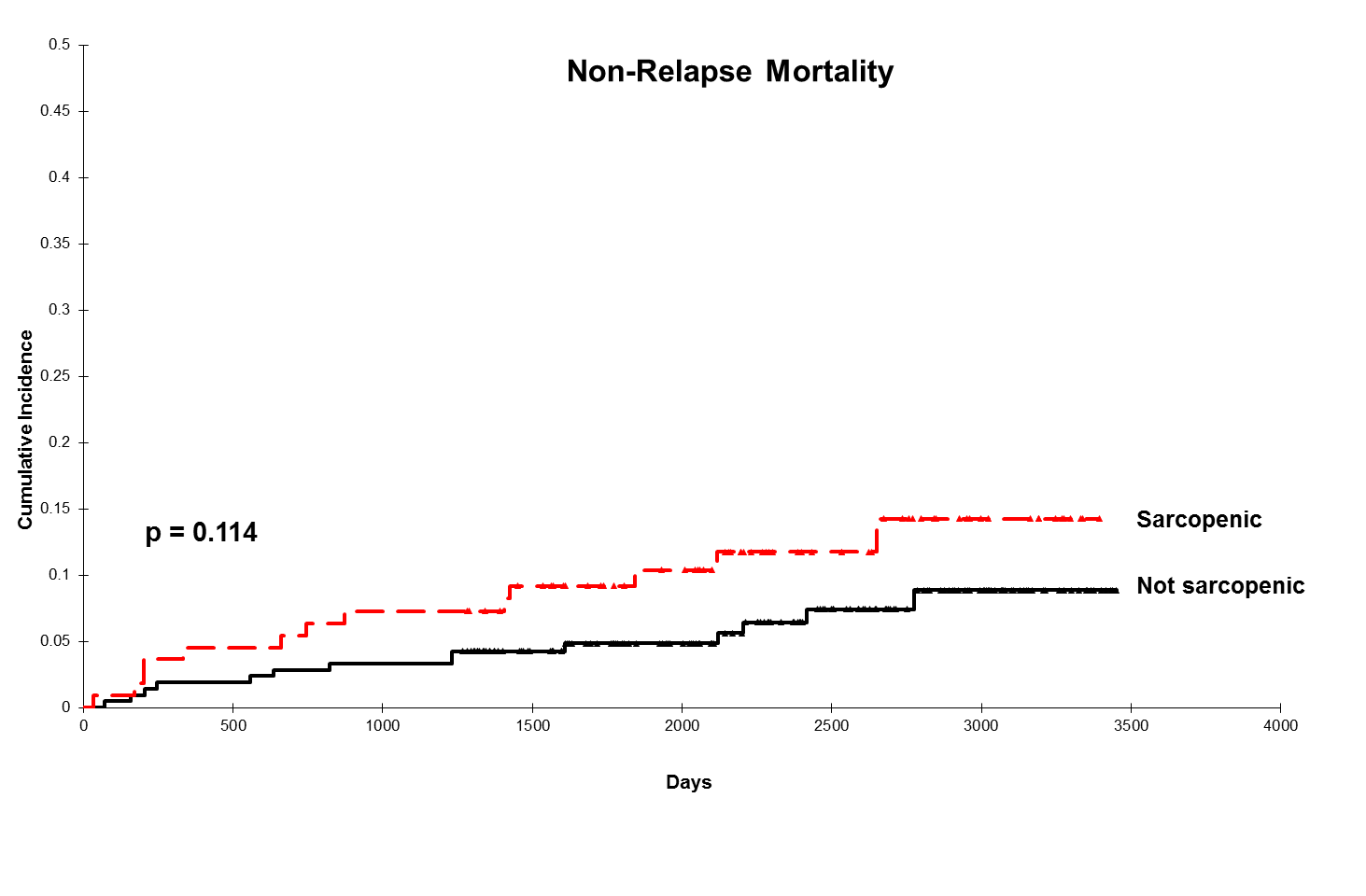

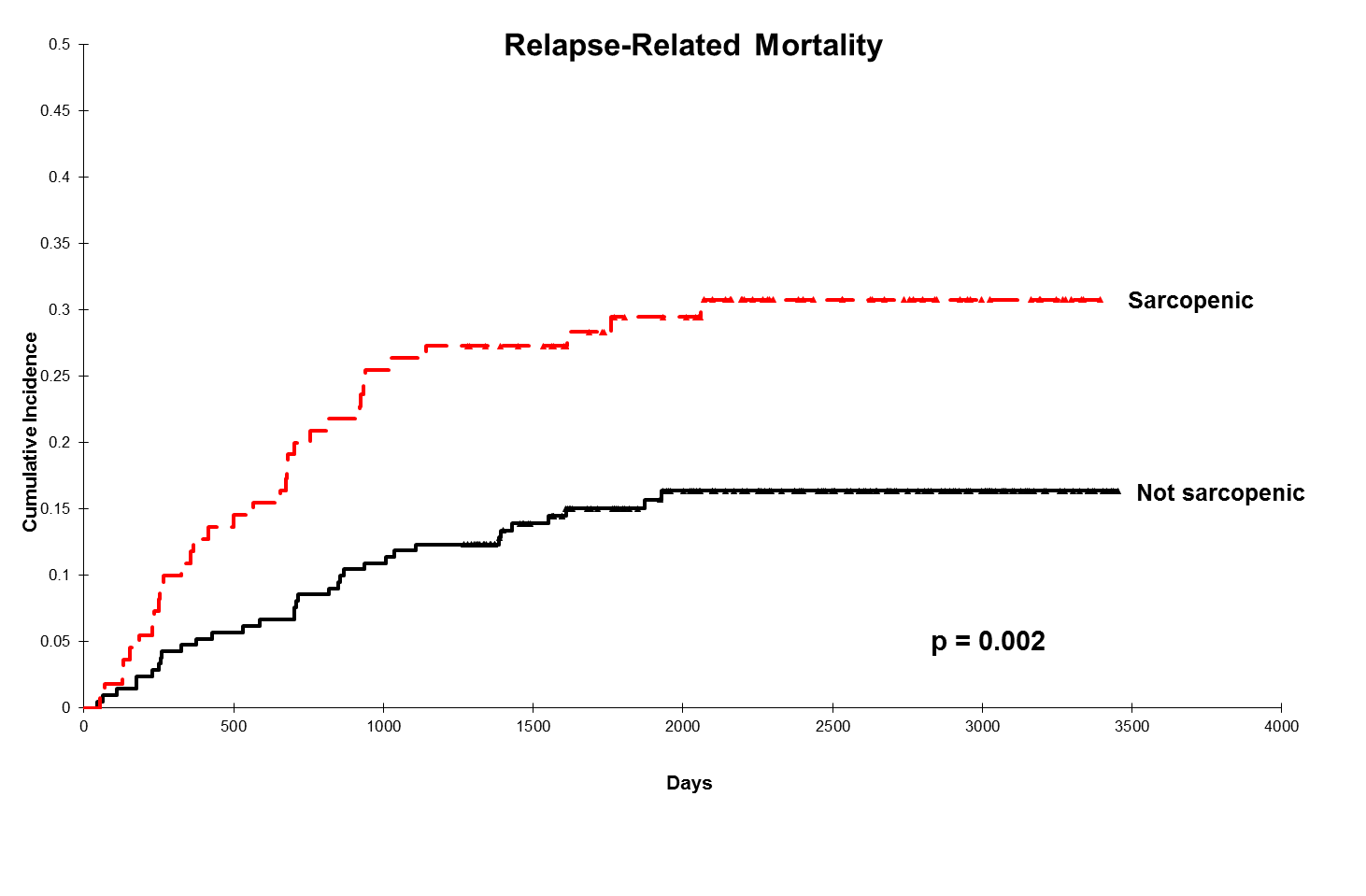


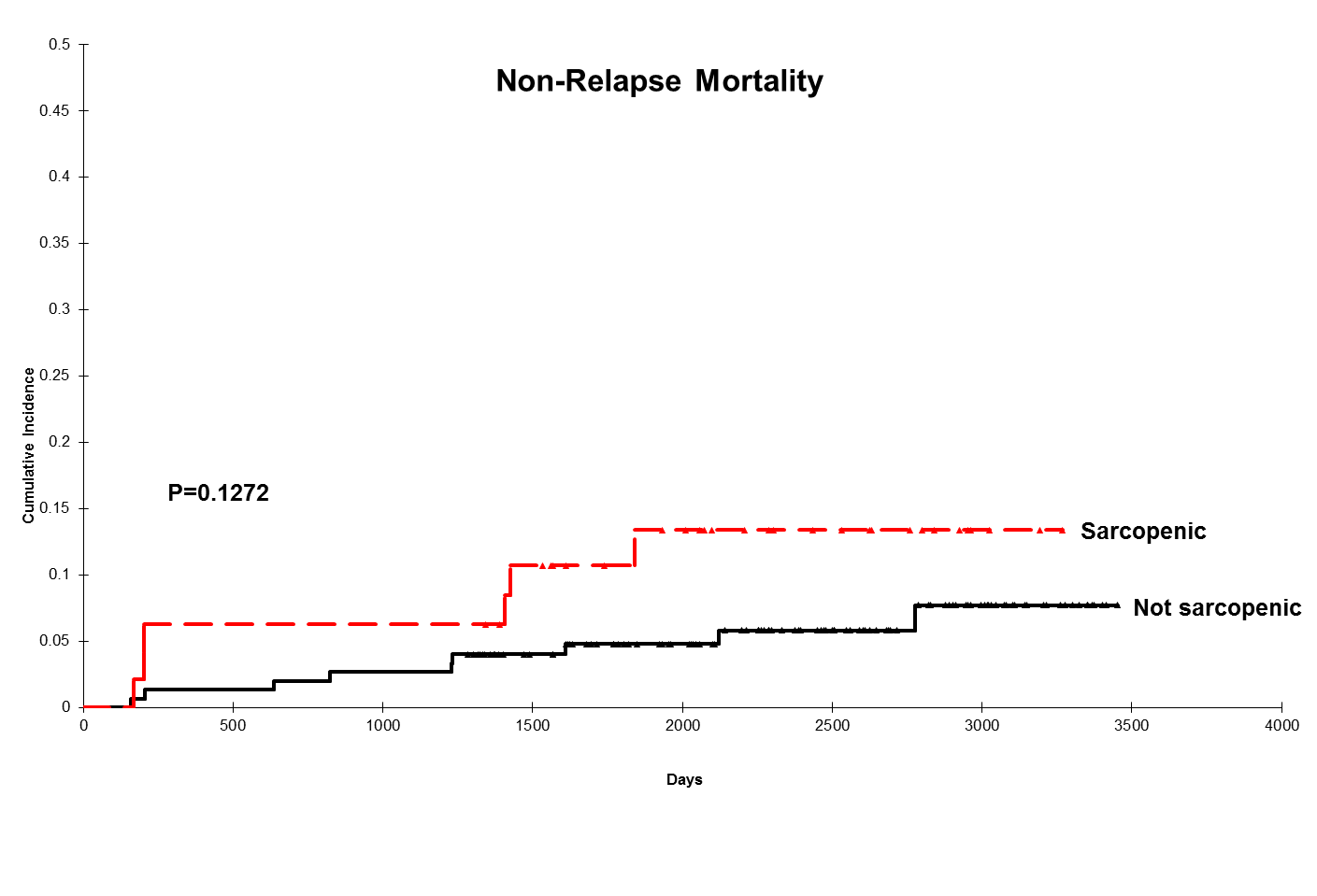

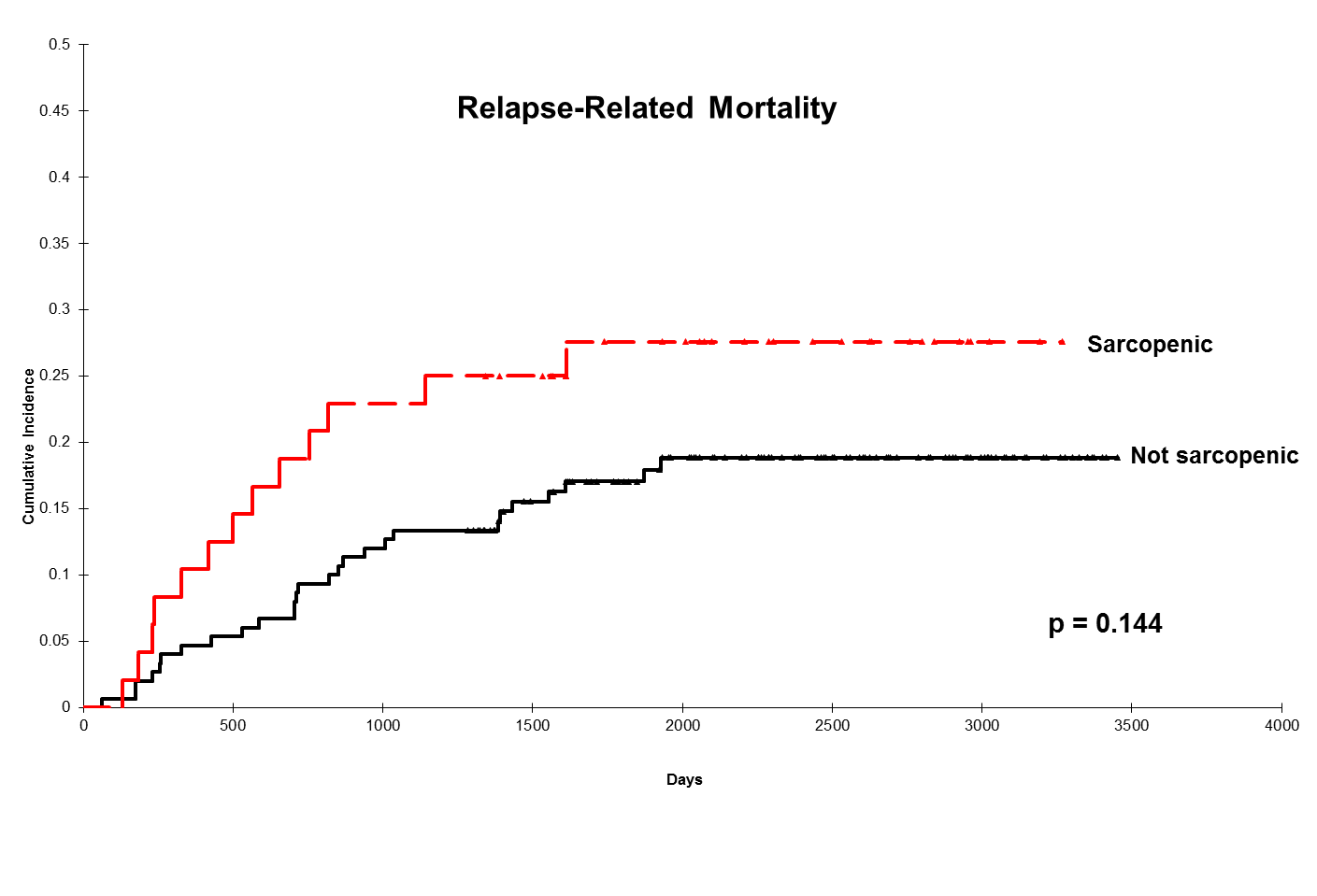
B.

**
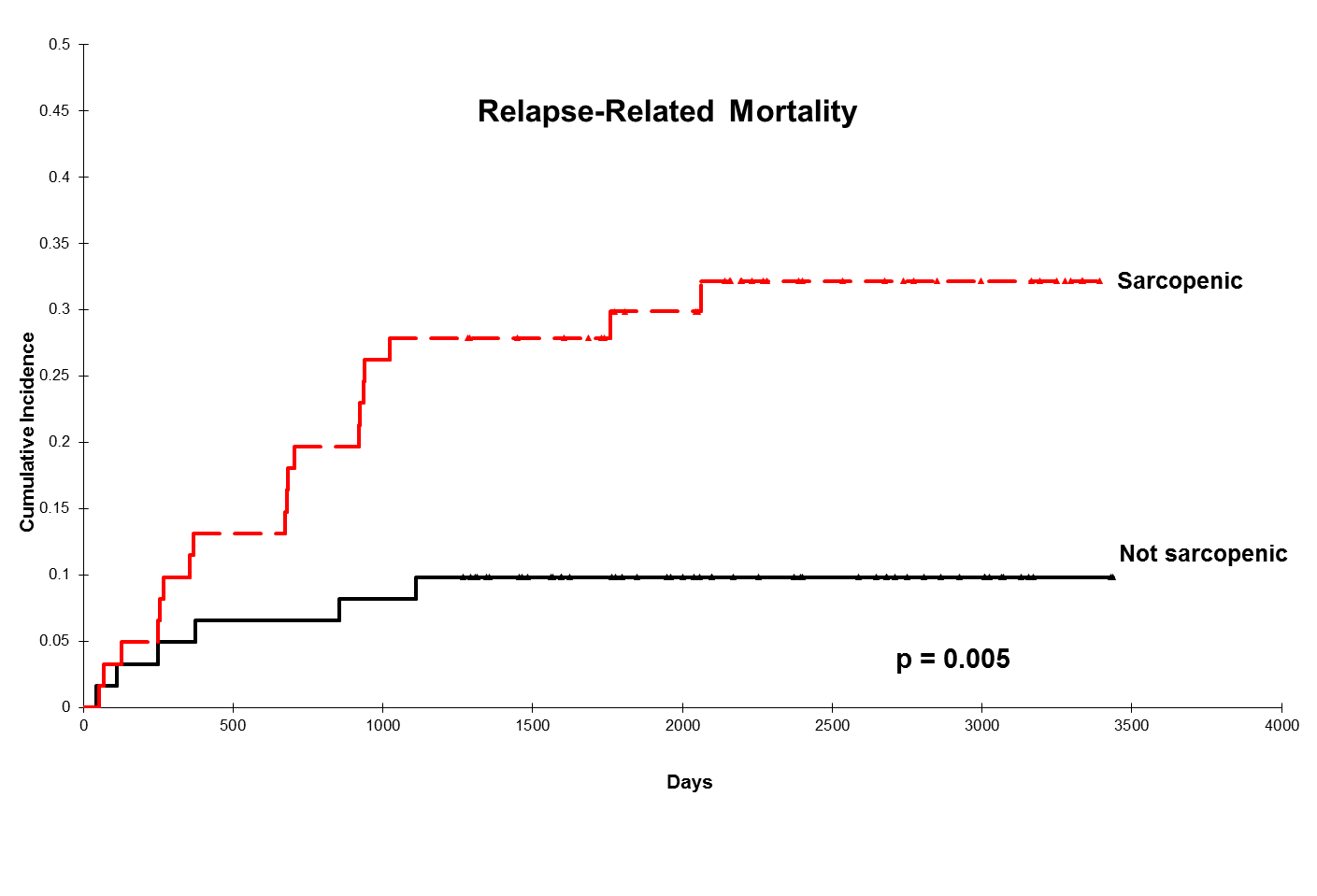

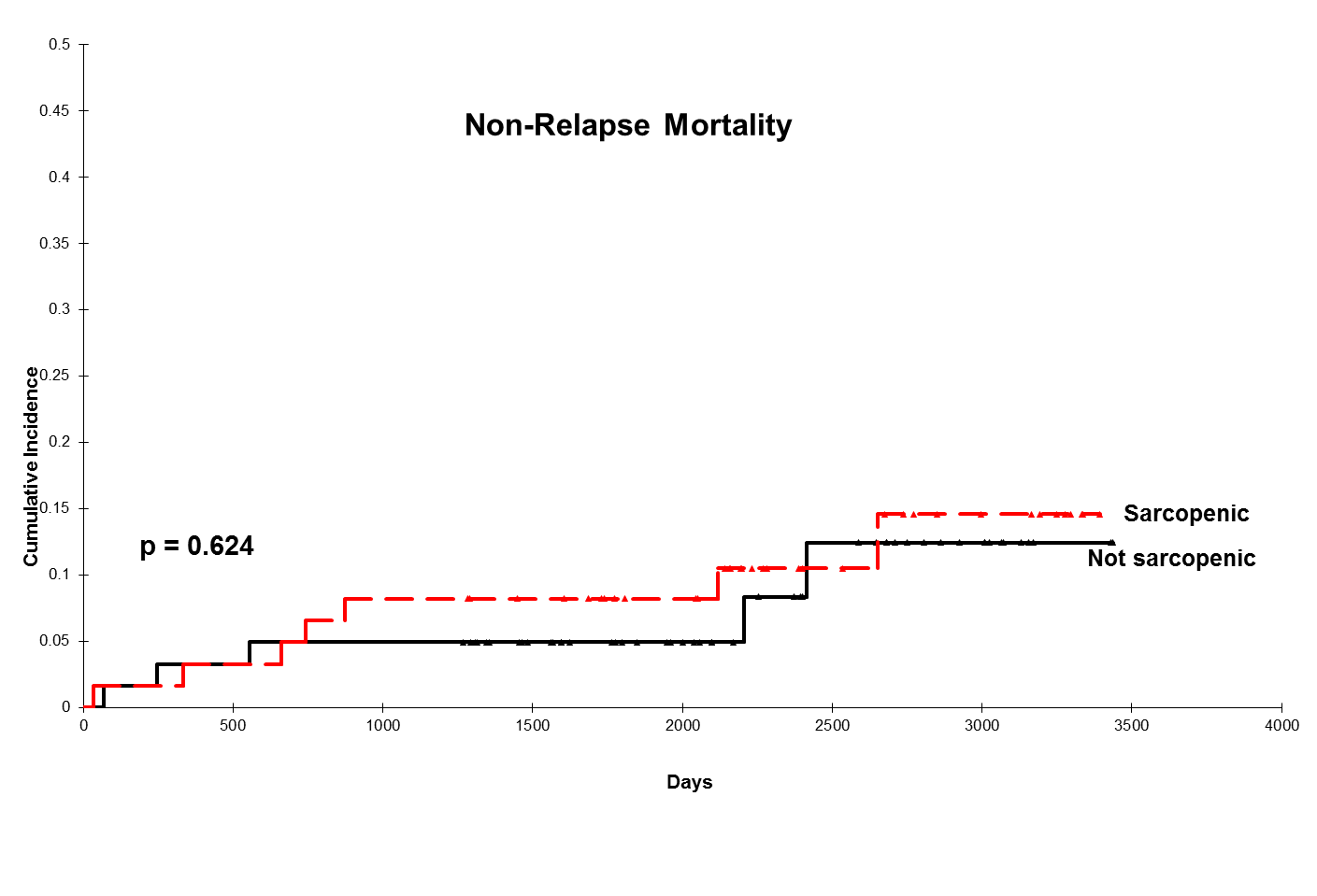
C.**

|  | **Body mass index** | **Skeletal muscle index** | **Visceral adipose tissue area** | **Subcutaneous adipose tissue area** | **Intramuscular adipose tissue area** |
| --- | --- | --- | --- | --- | --- |
| **Overall** |  |  |  |  |  |
| Weight (Kg) | 0.893 | 0.631 | 0.558 | 0.556 | 0.347 |
| Body mass index (Kg/m^2^) | - | 0.594 | 0.524 | 0.639 | 0.395 |
| Skeletal muscle index (cm/m^2^) | - | - | 0.400 | 0.251 | -0.036 |
| Visceral adipose tissue area (cm^2^) | - | - | - | 0.341 | 0.394 |
| Subcutaneous adipose tissue area (cm^2^) | - | - | - | - | 0.340 |
| **Male** |  |  |  |  |  |
| Weight (Kg) | 0.926 | 0.478 | 0.510 | 0.662 | 0.518 |
| Body mass index (Kg/m^2^) | - | 0.619 | 0.521 | 0.671 | 0.489 |
| Skeletal muscle index (cm/m^2^) | - | - | 0.271 | 0.362 | -0.043 |
| Visceral adipose tissue area (cm^2^) | - | - | - | 0.393 | 0.491 |
| Subcutaneous adipose tissue area (cm^2^) | - | - | - | - | 0.374 |
| **Female** |  |  |  |  |  |
| Weight (Kg) | 0.916 | 0.559 | 0.435 | 0.627 | 0.266 |
| Body mass index (Kg/m^2^) | - | 0.652 | 0.494 | 0.627 | 0.232 |
| Skeletal muscle index (cm/m^2^) | - | - | 0.227 | 0.414 | -0.183 |
| Visceral adipose tissue area (cm^2^) | - | - | - | 0.390 | 0.352 |
| Subcutaneous adipose tissue area (cm^2^) | - | - | - | - | 0.226 |

**Table S3.** Correlation between imaging parameters and conventional measures such as body weight and body mass index for the overall cohort and by sex

**Table S4.** Patient characteristics: individuals with and without post-HCT scans

| **Characteristics** | **Scan (N=275)** | **No Scan (N=45)** | **P-Value** |
| --- | --- | --- | --- |
| **Age at HCT** |  |  |  |
| **Mean, years (SD)** | 51.1 (13.2) | 49.0 (14.3) | 0.177 |
| **Race/Ethnicity, No. (%)** |  |  |  |
| Non-Hispanic white | 142 (51.6) | 30 (66.7) |  |
| Hispanic | 79 (28.7) | 6 (13.3) |  |
| Asian | 38 (13.8) | 7 (15.6) |  |
| Other | 16 (5.8) | 2 (4.4) | 0.152 |
| **Diagnosis, No. (%)** |  |  |  |
| Hodgkin lymphoma | 79 (28.7) | 5 (11.1) |  |
| Non-Hodgkin lymphoma | 150 (75.8) | 40 (88.9) | 0.013 |
| DLBCL | 106 (54.1) | 25 (62.5) |  |
| Mantle cell | 47 (24.0) | 3 (7.5) |  |
| Follicular | 19 (9.7) | 5 (12.5) |  |
| T-cell | 16 (8.2) | 5 (12.5) |  |
| Other | 8 (4.1) | 2 (5.0) | 0.222* |
| **Conditioning, No. (%)** |  |  |  |
| BEAM | 156 (56.7) | 27 (60.0) |  |
| CBV | 110 (40.0) | 16 (35.6) |  |
| Other | 9 (3.3) | 2 (4.4) | 0.810 |
| **Remission status at HCT, No. (%)** |  |  |  |
| Complete remission | 117 (42.5) | 18 (40.0) |  |
| Not in complete remission | 158 (57.5) | 27 (60.0) | 0.749 |
| **Karnofsky Performance Score, No. (%)** |  |  |  |
| >80 | 199 (72.4) | 32 (71.1) |  |
| ≤80 | 74 (26.9) | 12 (26.7) |  |
| Unknown | 2 (0.7) | 1 (2.2) | 0.982 |
| **HCT-Comorbidity Index, No. (%)** |  |  |  |
| 0-2 | 230 (83.6) | 38 (84.4) |  |
| ≥3 | 45 (16.4) | 7 (15.6) | 0.892 |

Abbreviations: HCT, hematopoietic cell transplantation; No., number; DLBCL, diffuse large B-cell lymphoma; BEAM, carmustine (BCNU), etoposide, cytarabine (ARA-C), melphalan; CBV, cyclophosphamide, BCNU, etoposide (VP-16);

*Subset analysis within non-Hodgkin lymphoma

**Table S5.** Pairwise t-test comparison of pre- and post-HCT measures

|  | **Overall cohort** | | | | **Male** | | | | **Female** | | | |
| --- | --- | --- | --- | --- | --- | --- | --- | --- | --- | --- | --- | --- |
|  | **Pre-HCT** | **Post-HCT** | **Diff** | ***P*-Value** | **Pre-HCT** | **Post-HCT** | **Diff** | ***P*-Value** | **Pre-HCT** | **Post-HCT** | **Diff** | ***P*-Value** |
| Body mass index, mean, kg/m^2^ (SD) | 28.4  (5.6) | 27.4  (5.4) | -0.8  (1.5) | <0.001 | 29.1  (5.1) | 28.4  (4.8) | 0.8  (1.3) | <0.001 | 26.7  (6.1) | 25.8  (6.1) | 0.8  (1.8) | <0.001 |
| Skeletal muscle index, mean, cm^2^ (SD) | 50.8  (10.4) | 49.2  (10.2) | -1.5  (3.4) | <0.001 | 56.1  (7.7) | 54.3  (8.0) | -1.8 (3.8) | <0.001 | 41.9  (8.0) | 40.7  (7.4) | -1.1  (2.4) | <0.001 |
| Visceral adipose tissue area, mean, cm^2^ (SD) | 122.3  (80.0) | 119.3  (77.0) | -2.9  (27.5) | 0.084 | 143.0  (84.2) | 139.8 (81.1) | -3.2  (29.0) | 0.157 | 88.3  (58.8) | 85.8 (55.9) | -2.5  (25.0) | 0.325 |
| Subcutaneous adipose tissue area, mean, cm^2^ (SD) | 249.8  (112.7) | 247.6  (119.1) | -2.2  (50.7) | 0.493 | 246.8  (105.0) | 245.8 (113.6) | -1.1  (51.9) | 0.801 | 254.9 (125.0) | 250.8 (128.1) | -4.1  (48.7) | 0.415 |
| Intramuscular adipose tissue area, mean, cm^2^ (SD) | 12.5  (9.4) | 12.4  (8.4) | -0.1  (4.9) | 0.695 | 12.0  (8.9) | 11.8  (7.1) | -0.2  (5.1) | 0.695 | 13.4  (10.1) | 13.3 (10.3) | -0.1  (4.6) | 0.903 |
| Total abdominal adiposity, mean, cm^2^ (SD) | 382.9  (162.5) | 377.2  (165.4) | -5.7  (61.8) | 0.144 | 400.9  (158.9) | 395.9 (162.4) | -5.0  (61.2) | 0.307 | 353.0 (164.7) | 346.1 (166.4) | -6.9  (63.0) | 0.291 |

**Table S6**. Multivariable regression analysis, predictors of *de novo* sarcopenia after HCT

| **Variable** | **Odds Ratio (95% Confidence interval)** |
| --- | --- |
| **Age at HCT, years** | 1.04 (0.99-1.09) |
| **Sex** |  |
| Male | 1.00 |
| Female | 0.65 (0.23-1.88) |
| **Race/Ethnicity, No. (%)** |  |
| Non-Hispanic white | 1.00 |
| Hispanic | 0.46 (1.58-1.35) |
| Asian | 1.87 (0.57-6.14) |
| Other | 0.38 (0.04-3.38) |
| **Diagnosis, No. (%)** |  |
| Hodgkin lymphoma | 1.00 |
| Non-Hodgkin lymphoma | 0.50 (0.16-1.50) |
| **Conditioning, No. (%)** |  |
| Non-BEAM | 1.00 |
| BEAM | 0.87 (0.32-2.34) |
| **Remission status at HCT, No. (%)** |  |
| Complete remission | 1.00 |
| Not in complete remission | 1.46 (0.58-3.70) |
| **Karnofsky Performance Score, No. (%)** |  |
| >80 | 1.00 |
| ≤80 | 2.47 (1.06-7.30) |
| **HCT-Comorbidity Index, No. (%)** |  |
| 0-2 | 1.0 |
| ≥3 | 1.1 (0.45-2.68) |

Abbreviations: HCT, hematopoietic cell transplantation; No., number; BEAM, carmustine (BCNU), etoposide, cytarabine (ARA-C), melphalan.
